# The relationship between glucocorticoid receptor polymorphisms, stressful life events, social support, and post-traumatic stress disorder

**DOI:** 10.1186/s12888-014-0232-9

**Published:** 2014-08-12

**Authors:** Yulong Lian, Jing Xiao, Qian Wang, Li Ning, Suzhen Guan, Hua Ge, Fuye Li, Jiwen Liu

**Affiliations:** Division of Occupational and Environmental Health, College of Public Health, Nantong University, Nantong, Jiangsu China; Division of Occupational and Environmental Health, College of Public Health, Xinjiang Medical University, Urumqi, Xinjiang China

**Keywords:** Post-traumatic stress disorder, Trauma, Genetics, Glucocorticoid receptor, Stressful life events

## Abstract

**Background:**

It is debatable whether or not glucocorticoid receptor (*GR*) polymorphisms moderate susceptibility to PTSD. Our objective was to examine the effects of stressful life events, social support, GR genotypes, and gene-environment interactions on the etiology of PTSD.

**Methods:**

Three tag single nucleotide polymorphisms, trauma events, stressful life events, and social support were assessed in 460 patients with PTSD and 1158 control subjects from a Chinese Han population. Gene–environment interactions were analyzed by generalized multifactor dimensionality reduction (GMDR).

**Results:**

Variation in *GR* at rs41423247 and rs258747, stressful life events, social support, and the number of traumatic events were each separately associated with the risk for PTSD. A gene–environment interaction among the polymorphisms, rs41423247 and rs258747, the number of traumatic events, stressful life events, and social support resulted in an increased risk for PTSD. High-risk individuals (a large number of traumatic events, G allele of rs258747 and rs41423247, high level stressful life events, and low social support) had a 3.26-fold increased risk of developing PTSD compared to low-risk individuals. The association was statistically significant in the sub-groups with and without childhood trauma.

**Conclusions:**

Our data support the notion that stressful life events, the number of trauma events, and social support may play a contributing role in the risk for PTSD by interacting with GR gene polymorphisms.

## Background

Post-traumatic stress disorder (PTSD) is an anxiety disorder which can develop as a result of exposure to a traumatic event. Although epidemiologic investigations have indicated that the majority of individuals exposed to traumatic stress will experience some kind of transient distress, only a relative minority of trauma-exposed individuals go on to develop PTSD [1,2]. This suggests that the heterogeneity of the effects of trauma could in part be mediated by several environmental risk factors, including pre-, peri-, and post-traumatic stressful life events [3-5]. The associations between PTSD and these stressful life events have been examined in several studies. For example, two prospective cohort studies involving the World Trade Center disaster showed that higher numbers of negative life events experienced before and after 9/11 were associated with PTSD among New York City adults and rescue, recovery, and clean-up employees on September 11, 2001 [6,7]. In several other prospective studies involving combat-related PTSD, pre-traumatic life events, such as multiple deployments, preparedness, unit cohesion, and post-traumatic life events (e.g., job loss, serious illness, and relationship and social difficulties) were predictors of PTSD onset [8-11]. However, social support is considered to be a protective buffer against the development of PTSD. Evidence from several cohort studies also confirmed that social support buffers the negative impact of exposure to trauma, and the presence and severity of the various PTSD symptoms may be moderated to some extent by social support [4,7,8]. These results seem to confirm that successive or cumulative exposure to stressful or adverse events weaken one’s coping ability, increase the stress -responsiveness of the HPA axis and psychological vulnerability to trauma, and therefore increases the risk for PTSD; perceived and embedded social support may influence an individual’s subjective appraisal and his/her negative internal or external responses to a traumatic event [12].

Apart from these environmental risk factors, genetic factors also play an important role in determining susceptibility among those who have experienced trauma. Twin and family studies have consistently shown that the heritability of PTSD is approximately 30-40% [13-15]. Candidate gene and genome-wide association studies have also implicated genes in the dopaminergic system, the hypothalamic-pituitary -adrenal (HPA) axis, the locus coeruleus-noradrenergic system, and those encoding neurotrophins directly regulating PTSD [16,17]. A recent review by Zoladz et al. [18] indicated a significant interaction of these genes with environmental factors, such as poor socioeconomic conditions, childhood adversity, low social support, and prior trauma that have previously been shown to significantly increase one’s likelihood of developing PTSD. The findings have revealed that specific genetic polymorphisms appear to increase PTSD susceptibility in individuals who experience the appropriate “disorder-triggering” situations.

The HPA axis is the major constituent of the neuroendocrine response to acute and chronic stress. Increased negative feedback of glucocorticoids (GCs) on the HPA axis is often considered as one of the hallmark biological correlates of PTSD, although cortisol alterations in PTSD are complex and somewhat inconsistent [19-21]. The increased sensitivity of the HPA axis, including altered baseline cortisol levels and an increased sensitivity of the GR, can also be induced by adulthood trauma exposure independent of PTSD [22,23]. The increased sensitivity of GRs and the receptor expression may be a consequence of specific genetic backgrounds associated with *GR* genotypes [24,25]. Although a number of studies have also shown that GR polymorphisms are associated with changes in GCs sensitivity or altered cortisol [24,26,27], few studies have indicated that PTSD is associated with the presence of single nucleotide polymorphisms (SNPs) in genes associated with *GR*. Individuals with PTSD after intensive care therapy who carried the homozygous G-allele of the BclI SNP (rs41423247) had more severe PTSD than non-carriers or heterozygous carriers [28]. Another study by Bachmann et al. [29] showed that the N363S and BclI *GR* polymorphism frequencies were not significantly more frequent between PTSD patients and control subjects. However, the findings are difficult to interpret due to the small number of subjects with PTSD and a BclI GG genotype in the study.

Additionally, one recent prospective study by van Zuiden et al. [24] examined military deployment representing pre-existing vulnerability factors for development of PTSD symptoms. Specifically, a significant interaction between the effect of the GR haplotype (BclI) and a high level of childhood trauma was associated with a higher *GR* number. Participants who carried the G allele and reported a high level of childhood trauma also appeared to be at increased risk for development of PTSD symptoms, although this effect failed to reach statistical significance. Clearly, further research is required to explain the basis for such disparate findings.

Although research has highlighted a role for both environmental factors, including trauma and stressful life events, and the *GR* gene in the development of PTSD, no investigations have specifically examined the role of the interaction between these environmental factors and *GR* gene polymorphisms in the etiology of PTSD. On the basis of the above data, we hypothesized that vulnerability to trauma events is accentuated by stressful life events, social support, and variation in *GR*, which may result in an increased risk for PTSD. We aimed to elucidate the following: i) the main effect of the *GR* gene, stressful life events, social support, and trauma on PTSD; ii) the interaction between *GR* polymorphisms associated with PTSD; and iii) the potential interaction among *GR* gene variants and these environmental factors on PTSD.

## Methods

### Subjects

Six hundred forty patients with PTSD were recruited from the Department of Psychiatry, First Hospital of Xinjiang Medical University and Xinjiang Mental Health Center, Xinjiang, China, between January 2011 and October 2012. A total of 2000 healthy subjects were also selected randomly from local community samples as controls. All the subjects were of Chinese Han origin and unrelated inhabitants of northern China. All patients were interviewed by at least two trained psychiatrists using the Diagnostic and Statistical Manual of Mental Disorders, Fifth Edition (DSM-V) criteria for PTSD [30]. To meet PTSD criteria, these patients satisfied (1) DSM-V Criterion A event, (2) at least one symptom of Criterion B, (3) at least one symptom of Criterion C,(4) at least three symptoms of Criterion D, (5) at least three symptoms of Criterion E, (6) duration of at least one month (symptoms in Criteria B, C, D and E), and (7) significant distress or impaired functioning in their personal life, relationships, work or school due to these symptoms. The patients with the following conditions were excluded, including a history of head trauma, organic brain syndrome, psychosis or bipolar disorder, or dependence on substances other than alcohol and nicotine. All the control subjects underwent structured clinical interviews to exclude those who had any current or previous psychiatric disorders, or who had a family history of psychiatric or neurological disorders, or who were alcoholics or drug abusers. Because the purpose of the present study was to explore the factors affecting the risk for PTSD when individuals face traumatic events, it was determined whether or not all of the control subjects had witnessed or experienced traumatic life events using the Chinese Traumatic Life Events Questionnaire (TLEQ). Respondents who had no traumatic experiences were excluded; finally, 1158 subjects were interviewed for the study. The description of the case and control groups is shown in Table 1. All participants provided written informed consent. The study was approved by the Ethics Committee for Xinjiang Medical University and Xinjiang Mental Health Center.

**Table 1:** **Demographics and factors associated with PTSD**

**Variable**	**PTSD (n = 460)**	**Controls (n = 1158)**	**t/x** ^**2**^	**OR**	**95% CI**	**P**
Age (mean, SD)	37.87(9.85)	34.47(10.84)	4.10			<0.001
Gender (n, %)						
Male	253(55.0)	662(39.9)	0.63			>0.05
Female	207(45.0)	496(60.1)				
Stressful life event* (n, %)						
Severe	376(81.8)	630(54.4)	52.79	3.76	2.04,6.71	<0.001
Moderate	84(18.2)	528(45.6)		1.00		
Social support* (n, %)						
Lower	271(58.9)	252(21.8)		1.00		
Moderate	155(33.7)	614(53.0)	109.36	0.11	0.06,0.19	<0.001
Higher	34(7.4)	292(25.2)		0.45	0.25,0.79	
The number of Trauma events* (n, %)						
0-1	115(25.0)	486(42.0)		1.00		
2-5	115(25.0)	434(37.5)	203.07	1.70	0.80,3.58	<0.001
6-9	211(45.9)	226(19.5)		5.98	2.82,12.67	
≥10	19(4.1)	12(1.0)		6.69	3.16,14.18	
Type of trauma (n, %)						
Natural disaster	22(4.8)	68(5.9)				
Motor vehicle accident	46(10.0)	165(14.3)				
Other accident (e.g., workplace accident)	50(10.9)	102(8.8)				
Serious illness (e.g., HIV, cancer)	64(13.9)	198(17.1)				
Death of loved one	106(23.0)	207(17.9)				
Injury/illness of loved one	70(15.2)	101(8.7)	109.81			<0.001
Witness family violence	84(18.3)	135(11.6)				
Childhood physical assault	46(10.0)	72(6.2)				
Robbed with a weapon	22(4.8)	23(2.0)				
Adult physical assault (without weapon)	28(6.1)	50(4.3)				
Intimate partner violence	80(17.4)	94(8.1)				
Witness physical assault	66(14.3)	94(8.1)				
Threatened with physical assault	40(8.7)	55(4.8)				
Childhood sexual assault by adult	32(7.0)	66(5.7)				
Childhood sexual assault by peer	40(8.7)	92(7.9)				
Young adult sexual assault	32(7.0)	41(3.5)				
Adult sexual assault	44(9.6)	46(4.0)				
Unwanted sexual attention	10(2.2)	10(0.8)				
Stalking	4(0.9)	15(1.3)				
Miscarriage (self or partner)	10(2.2)	18(1.6)				
Abortion (self or partner)	24(5.2)	60(5.2)				
Other (e.g., finding dead bodies, combat, violent death of pet, witness drug overdose of stranger)	20(4.3)	53(4.5)				

*adjusted for age.

### Trauma exposure

The Chinese TLEQ was used to assess the history of exposure to 22 different types of traumatic experiences that meet the DSM-IV PTSD criterion A1 definition of trauma [31]. The traumatic events include childhood physical and sexual abuse, combat exposure, motor vehicle accidents, natural disasters, and adult sexual and physical assault. The measure also assesses whether or not the experience meets the DSM-IV PTSD criterion A2 and asks the respondent to indicate the number of times each event occurred on a 7-point scale ranging from “never” to “more than 5times”. Participants also identified their “worst” trauma from the list of trauma types included on the TLEQ. The TLEQ has shown good test–retest reliability and predictive validity with respect to PTSD diagnoses [31,32]. The number of traumatic events experienced was categorized into quintiles of approximately equal size (1, 2 to 5, 6 to 9, and ≥10) to facilitate further analysis. Participants were asked whether, by age 13, they had witnessed or experienced a violent crime, had been sexually abused, or had been physically abused. Endorsement of any of these adverse childhood experiences was coded as positive for exposure to childhood trauma.

### Stressful life events

Stressful life events were assessed using the life events scale (LES) developed by Yang and Zhang [33], in which a total of 48 items are classified into 3 categories, including family life (28 items), work problems (7 items), social interactions and other aspects (7 items). The LES has been widely used in a Chinese population. These events encompass serious illnesses, housing, relationship, and social difficulties, relationship breakups, unemployment, and financial crisis. This scale indicates that change in one’s life requires an effort to adapt, then an effort to regain stability. The questionnaire has demonstrated high reliability and validity and was widely used in Chinese social research [34,35]. This scale assessed the following four aspects of negative life events: the time of occurrence of life events (absent = 1, 1 year earlier = 2, within the last 1 year = 3, chronicity = 4); character of life events (good = 1, bad = 2); influence of mind (absent = 1, mild = 2, moderate = 3, severe = 4, extreme = 5); and duration of influence (≤3 months = 1, 3–6 months = 2, 6–12 months = 3, >12 months = 4). The 75% percentile (a score of 32) in the sample was used as a cut-off value for grouping the moderate and severe levels of stressful life events [35].

### Social support

Social support was measured with the Chinese Social Support Rating Scale (SSRC) [36], which consists of a total of 10 items with 3dimensions (objective support [three items], subjective support [four items], and the use of social support [three items]). The Crohnbach’s *α* coefficient was 0.81 and test-retest reliability was 0.92 [36]. Each of the 10 items was classified into four grades, ranging from completely disagree (1 point) to completely agree (4 points). Therefore, the higher the scores were, the higher the degree of social support. A total score <33 characterizes low social support, 33–45 equates to moderate social support level, and scores >45 equate to high social support [36,37].

### Marker selection

SNPs within the *GR* gene were selected from dbSNP (http://www.ncbi.nlm.nih.gov/projects/SNP/) and the HapMap database (http://www.hapmap.org), of which the tagSNPs were defined by Haploview (version 4.0). The criteria for tagging were a linkage disequilibrium (LD) threshold (r^2^) ≥ 0.8 and a minor allele frequency (MAF) ≥ 0.1. According to the most recent HapMap build (HapMapRel28August10 Build) among the Han Chinese in Beijing (CHB) population, 21 tagSNPs were selected. We further screened these tagSNPs on the basis of the reported role in central regulation of the HPA-axis and PTSD phenotypes [27-29]. Finally, only three taqSNPs (rs258747, rs41423247, and rs10482605) were selected for genotyping.

### DNA sampling and genotyping

Genomic DNA was extracted from peripheral blood leukocytes using a Genomic DNA extraction kit. Genotyping was performed using multiplex SNaPshot technology with an ABI fluorescence-based assay discrimination method (Applied Biosystems, Foster City, CA, USA). The multiplex SNaPshot detection of single-base extended probe primers was based on fluorescence and extended length detected by capillary electrophoresis on an ABI3130XL Sequencer (Applied Biosystems). Six primers for SNaPshot extension reactions were designed by Primer3 online software (v.0.4.0; http://frodo.wi.mit.edu/primer3/) according to the reference sequences from dbSNP (http://www.ncbi.nlm.nih.gov/SNP). For quality control, a random sample of 5% of the cases and controls was genotyped twice by different researchers, with a reproducibility of 100%. The minor allele counts were compared with database (http://www.ncbi.nlm.nih.gov/projects/SNP) and the data were matched well. Genotyping was performed blind to group status.

### Statistical analysis

All genotyped SNPs were examined using chi-square (χ^2^) goodness-of-fit test for genotypic distributions in Hardy-Weinberg equilibrium (HWE). The age of participants was analyzed as a continuous variable and tested using a t-test. The genotypic and allelic frequencies between the patient and control groups were compared by Pearson (χ^2^) using SPSS for Windows (version17.0; SPSS, Inc., Chicago, IL, USA). Logistic regression models were used to examine the association between PTSD, stressful life events, and social support with adjustment for age. The linkage disequilibrium analysis between the patient and control groups was compared using the UNPHASED program (Dudbridge, 2003). In addition, gene–gene and gene–environment (GxE) interactions were analyzed by multifactor dimensionality reduction software (MDR 2.0 beta 6), which is a free open source accessible at http://sourceforge.net/projects/mdr/. Previous studies showed that age may affect GR genetic effect on PTSD susceptibility; we included age as covariates in our GxE interaction analyses. All interactions were tested using 10-fold cross-validations in an exhaustive search considering all possible combinations. The model with the highest testing balance accuracy and cross-validation consistency was selected as the ‘best model.’ Statistical significance was determined using a 1000-fold permutation test. To clarify the GxE results, we computed the (χ^2^) and odds ratios (ORs) with 95% confidence intervals (CIs) to determine the set of risk factors. The significance level for all statistical tests was set at a corrected P-value of 0.05.

## Results

The genotypic distributions of the three polymorphisms were all in HWE in the case and control groups, which suggests that no particular genetic selection of the study groups occurred. Allele frequencies in our sample were similar to those reported for Caucasians in HapMap except rs10482605 (MAF = 0.029).

The basic clinical characteristics of participants are shown in Table 1; the mean (SD) age of cases and controls was 37.87 (9.85) years and 34.47 (10.84) years, respectively. The most common event for PTSD patients was death of loved one (23.0%) followed by childhood sexual assault by adult (20.0%). The most common event in control group was death of loved one (17.9%). After adjustment for age, severe stressful life events were significantly associated with risk for PTSD. The OR of the effect of stressful life events on the risk of PTSD was 3.76 (95% CI = 2.04-6.71). Additionally, higher and moderate social support were protective for PTSD compared with lower social support with an OR of 0.45 (95% CI = 0.25-0.79) and 0.11 (95% CI = 0.06-0.19), respectively. It was also expected that the likelihood of developing PTSD increased as a function of the number of reported traumatic events. Individuals who had experienced > 5 traumatic events were 5-fold more likely to develop PTSD than those who had experienced ≤ 5 events.

Logistic regression analysis was conducted to test the associations between the three polymorphisms (rs10482605, rs258747, and rs41423247) and PTSD after adjusting for age. Significant differences in allele and genotype frequencies were detected between healthy control subjects and PTSD patients. As shown in Table 2, we demonstrated that the presence of GG and GA genotypes and the G allele (rs258747) significantly reduced the risk of PTSD, while presence of the A allele and AA genotype was a positive factor with respect to disease risk. Subjects with the G allele and CG genotype (rs41423247) had an increased likelihood of PTSD compared with those with the C allele and CC genotype (OR = 1.29, 95% CI = 1.09-1.54). The rs10482605 is not associated with PTSD. In addition, we measured linkage disequilibrium by D^’^ and R^2^ between the three polymorphisms using the UNPHASED program, which showed that the three polymorphism sites were not likely to be linked (R^2^ < 0.8).

**Table 2:** **Association of genotype frequencies of the glucocorticoid receptor gene in patients with PTSD compared with controls when adjusted for age**

**SNPs**	**PTSD (n, %)**	**Controls (n, %)**	**x** ^**2**^	**OR**	**95% CI**	**p**
rs10482605						
T/T	428(93.1)	1105(95.4)	4.68	1.0		0.096
C/T + C/C	32(6.9)	53(4.6)		0.66	0.43,1.01	
C	49(5.3)	46(2.0)	3.92	1.0		0.087
T	871(94.7)	2270(98.0)		0.36	0.24,1.05	
rs41423247						
C/C	230(50.0)	684(59.1)	11.14	1.0		
C/G	202(43.9)	412(35.6)		1.46	1.16,1.83	0.004
G/G	28(6.1)	62(5.3)		1.34	0.84,2.15	
C	662(72.0)	1780(76.9)	8.54	1.0		0.003
G	258(28.0)	536(23.1)		1.29	1.09,1.54	
rs258747						
A/A	190(41.3)	562(48.5)	11.77	1.0		
G/A	200(43.5)	480(41.5)		1.23	0.97,1.56	0.003
G/G	70(15.2)	116(10.0)		1.78	1.27,2.51	
A	580(63.0)	1604(69.3)	11.59	1.0		0.001
G	340(37.0)	712(30.7)		1.32	1.13,1.55	

The results of exhaustive MDR analysis evaluating the combinations of the three tested SNPs are summarized in Table 3. The best combination for a potential interactive polymorphism in predicting PTSD was the rs258747 and rs41423247 combination. The testing balance accuracy for this two-locus model was 51.72% and cross-validation consistency was 10/10 (100%); an empiric P-value of < 0.01 based on 1000-fold permutations was obtained.

**Table 3:** **MDR analyses on gene–gene interactions in PTSD patients and controls**

**Mode**	**Test accuracy**	**OR**	**95% CI**	**Cross-validation consistency**	**P**
Rs258747	0.5125	3.23	1.84,5.67	7/10	<0.001
Rs258747, rs41423247	0.5172	5.47	2.61,11.45	10/10	<0.001

To explore the potential gene-environment interactions on PTSD, MDR analysis was used to search the association of the two SNPs with stressful life events, number of traumatic events, and social support. In the three-way gene-environment interaction model, the combination of stressful life events, traumatic events, social support, rs258747, and rs41423247 had the highest testing balance accuracy (51.72%) and cross-validation consistency (10/10) on the prediction of PTSD (Table 4). This suggests that highly stressful life events, a large number of traumatic events, and low social support under specific genotypes of the *GR* gene may increase the risk for PTSD. Although MDR analysis was able to identify the presence of an association among several factors, the results did not show detailed information about the interactions. We then used logistic regression analysis to determine the set of risk factors selected by MDR. The participants were divided into the following three groups: 1) high risk were those with a G allele (rs258747), G allele (rs41423247), highly stressful life events, a large number of traumatic events, and low social support; 2) low risk were those with an A allele (rs258747), C allele (rs41423247), moderate stressful life events, a small number of traumatic events, and high social support; and 3) medium risk was others. As shown in Table 5, adults in the high- (OR = 3.26, 95% CI = 2.46-4.32) and medium-risk groups (OR ranged from 2.44 to 2.94) were significantly more likely to have PTSD that those in the low-risk group. Given the reported significant association between the timing of trauma exposure and PTSD in previous studies, we further analyzed the above interaction according to the timing of trauma exposure. Our population was divided into persons with childhood trauma and without childhood trauma. The results remained significant, and the odds ratios very similar to those reported in the total population, as follows: the high-risk group who experienced childhood trauma (OR = 3.33, 95% CI =1.99-5.57); and the high-risk group who had no childhood trauma exposure (OR = 3.26, 95% CI =2.32-4.57).

**Table 4:** **MDR analyses on gene–environmental interactions in PTSD patients and controls**

**Model**	**Test accuracy**	**OR**	**95% CI**	**Cross-validation consistency**	**P**
Rs 41423247, stressful life events, number of traumatic events, social support	0.6491	4.06	2.76,5.98	10/10	<0.001
Rs258747, rs41423247, stressful life events, number of traumatic events, social support	0.6586	4.17	2.87,6.05	10/10	<0.001

**Table 5:** **Interaction effect of the glucocorticoid receptor gene, number of traumatic events, stressful life events, and social support for risk of PTSD when adjusted for age**

**Variables**	**Total**			**With childhood trauma**			**Without childhood trauma**		
	**n**	**PTSD %**	**OR (95% CI)**	**n**	**PTSD %**	**OR (95% CI)**	**n**	**PTSD %**	**OR (95% CI)**
A-C-ML-LT-MS	778	14.7	1.00	161	20.5	1.00	617	13.1	1.00
G-G-HL-HT-LS	418	35.9	3.26(2.46,4.32)^a^	130	46.2	3.33(1.99,5.57)^a^	288	33.7	3.26(2.32,4.57)^a^
A-C-HL-HT-LS	948	33.5	2.94(2.31,3.73)^a^	227	38.2	2.40(1.49,3.85)^a^	721	30.4	2.89(2.18,3.83)^a^
A-G-HL-LT-MS	474	29.5	2.44(1.84,3.25)^a^	52	36.5	2.23(1.13.4.42)^a^	422	30.3	2.88(2.11,3.94)^a^
G-G-ML-LT-LS	330	30.9	2.61(1.92,3.55)^a^	29	41.4	2.74(1.19,6.29)^a^	301	29.2	2.73(1.94,3.85)^a^
All Others^a^	288	33.3	2.91(2.12,3.99)^a^	57	44.6	3.13(1.63,5.98)^a^	231	30.6	2.92(2.03,4.20)^a^

ML moderate level stressful life events, HL high level stressful life events, HT high number of traumatic events (more than five traumatic events), LT low number of traumatic events (five or fewer events), MS moderate social support or high social support, LS low social support.

^a^P<0.05.

## Discussion

In this study, we explored the main and interaction effects of the *GR* gene, traumatic events, stressful life events, and social support on the risk for PTSD. Exposure to highly stressful life events, a large number of traumatic events, and low social support each independently increases the risk for PTSD on exposure to trauma. These results are consistent with the findings of Kolassa et al. [38] and Huang et al. [32], who showed that higher numbers of different lifetime traumatic event types led to a higher prevalence of lifetime PTSD in a dose–response relationship, and individuals who had childhood adversity and adult traumatic events were more likely to develop lifetime PTSD compared with those who experienced either type of adverse event. Our study also confirmed the important role of social support in buffering or aggravating the effects of trauma events and other environmental stressors. Our study further suggests that this risk is heightened among individuals with risk alleles of the two SNPs (rs258747 and rs41423247) of the *GR* gene. Strong association between the combination of the two SNPs and PTSD in this study gives further evidence in support of the functional role of the *GR* gene in the development of PTSD. The rs41423247 polymorphism might affect the *GR* gene promoter by selectively acting on repressor or enhancer sites within the promoter, thereby altering GC sensitivity [39]. Previous studies have reported an association between SNP and increased sensitivity to glucocorticoids following a DEX-CRH test, a lower cortisol response after psychological stress [40,41], and more traumatic memories and major depressive disorders [28,42]. Hauer et al. [28] observed that individuals who were homozygous carriers of the minor (G) allele of the SNP had more traumatic memories and lower plasma cortisol levels, and were at increased risk for development of PTSD symptoms after cardiac surgery, while homozygous G allele carriers had enhanced GR sensitivity and an increased number of GRs, which was a strong predictor of the presence of PTSD symptoms. In addition, Wüst et al. [43] showed strong genetic effects of the SNP variant on HPA-axis responsiveness in a male twin cohort. The SNP heterozygotes had high responses, both for cortisol and ACTH, following a psychological challenge than “wt-type” individuals. The altered baseline cortisol levels and increased sensitivity of the *GR* could reflect a pre-existing vulnerability, which increases the probability of developing PTSD following trauma exposure [23].

The rs10482605 SNP is a T/C polymorphism located in the promoter region of alternative exon 1C. The minor C allele (rs10482605) has lower transcriptional activity under unstimulated and different stimulation conditions (dexamethasone, forskolin, and phorbol 12-myristate acetate) in vitro, and the haplotype between rs10482605 and rs6198 leads to relative GC resistance and increased GRbeta mRNA stability, which may significantly contribute to individual vulnerability for the development of major depression [27]. Kumsta et al. [44] reported that the variant C allele (rs10482605) showed reduced transcriptional activity in a reporter gene assay, and the C allele was also associated with recurrent major depression in a Belgian study [45], thus suggesting possible functional effects of this SNP. There are no published data on the relationship between the SNP and PTSD. We did not observe a significant association between the SNP and the risk of PTSD in our Chinese Han sample, consistent with a previous study [46], in which no relationship between rs10482605 and cortisol responses to psychosocial stress was found in a child psychiatric population. In addition, the frequency of the minor allele of the SNP in the present study (0.029) was lower than that among other African (0.075), Caucasian (0.183), Belgian (0.013), Swedish (0.090), and Mexican populations ((0.133); [45,47]). The ethnic differences in polymorphism frequencies might contribute to inconsistent results in genetic association studies. Thus, we speculate that the polymorphism may not be involved in the pathophysiology of PTSD in the Chinese Han populations.

The rs258747 polymorphism is located in the 3′-flanking region, which is responsible for mRNA stabilization. Although no previous data have been published thus far in regard to PTSD, it has been reported recently that the variant is associated with the response to duloxetine in major depressive disorder and the response to lamotrigine in bipolar depression disorder [48]. We also found the rs258747 SNP is associated with PTSD. Moreover, rs258747 has been chosen as a tag SNP for the NR3C1 gene region and located in the large haplotype block [47]. The possible functional impact of the rs258747 polymorphism on NR3C1 function remains unsolved; however, it cannot be excluded that the SNP is located within the sequence involved in gene regulation. The other possibility is that this SNP is in strong linkage disequilibrium with other, functionally relevant variants causative for PTSD development. Our finding may also support the importance of the interactions between the two SNPs in the development of PTSD. The potential functional consequences of the investigated SNPs need further studies to understand the genetic factors underlying PTSD, as well as possible interactions between the *GR* gene and other plausible candidate genes.

The main finding of our work was that the *GR* variants interact with the number of traumatic and stressful life events and social support to predict PTSD; thus, the effect of trauma is different depending upon two SNPs (rs258747 and rs41423247), stressful life events, social support, and the number of traumatic events. The individuals with the G allele of *GR* rs258747, C allele of *GR* rs41423247, high level stressful life events, large number of traumatic events (>5 traumatic events), and low social support were at high risk for the development of PTSD. After dividing the trauma exposure into childhood trauma and non-childhood trauma, the GxE interaction remained significant. This finding is partially consistent with prior studies that have shown the *GR* genotype to be associated with risk for PTSD. A randomized controlled trial study by Hauer et al. [49] showed that a pre-operatively administered minimal cognitive behavioral intervention targeted to homozygous carriers of the G high-risk allele of the SNP rs41423247 reduces traumatic memories and post-traumatic stress disorder symptoms after heart surgery. In Dutch military personnel, the presence of the G allele of the SNP rs41423247, in combination with a high level of childhood trauma, was associated with a high GR number in PBMCs prior to deployment, which was identified to be a vulnerability factor for PTSD [26]. A prenatal GxE interaction of *GR* rs41423247 with maternal psychological symptoms could result in an increased risk of child HPA-axis dysfunction [50]. Although several previous studies highlight the importance of considering the GxE interaction when exploring the etiology of PTSD, few studies have reported other environmental factors in considering GxExE effects on the risk of PTSD, with the exception of the nature of trauma. Our findings give further evidence suggesting that HPA-axis related gene polymorphisms may modify the association between environmental factors and PTSD exposure to trauma. Therefore, future studies should be enhanced to explore not only the GxE relationship, but also the more complex GxGxE and GxExE for understanding genetics and environmental influences on PTSD.

In comparison with these previous studies, the current study had several strengths that deserve mention. Instead of using PTSD symptoms as an outcome, participants were directly interviewed by trained interviewers, resulting in a reliable PTSD diagnosis. In addition, we examined a range of lifetime traumatic events with a reliable and validated instrument. These events covered most, but not all, trauma types known to cause PTSD .Nevertheless, interpretation of this study should consider the following limitation. The subjects in our study were all from the Chinese Han population; we cannot adequately generalize from these results to populations with different racial backgrounds. Thus, the findings will require further replication in other population with a longitudinal cohort study. Stressful life events, social support and childhood trauma data were collected retrospectively without using a structured interview, which may have resulted in recall bias. Although we analyzed the gene-environment interactions on the risk of PTSD in groups with childhood exposure and non-childhood exposure, the more complex GxExE including childhood trauma or other kind trauma events was not examined due to the small sample size. In the present study, we investigated only three tag SNPs; therefore, it is possible that other polymorphisms of the genes may be more important in determining susceptibility to PTSD. Future studies of this gene would also be strengthened by additional mapping and sequencing to identify functional variants, as well as gene expression studies.

## Conclusions

We found evidence for a gene-environment interaction involving *GR* polymorphisms with the number of traumatic and stressful life events and social support, resulting in an increased risk of PTSD. These findings emphasize the potential cumulative effect of genetics and environmental factors on the probability of developing PTSD, and give further insight in possible mechanisms accounting for the differences in the individual vulnerability to PTSD.

## References

[1] Galea S, Ahern J, Resnick H, Kilpatrick D, Bucuvalas M, Gold J, Vlahov D (2002). Psychological sequelae of the September 11 terrorist attacks in New York City. N Engl J Med.

[2] Kessler RC, Sonnega A, Bromet E, Hughes M, Nelson CB (1995). Posttraumatic stress disorder in the National Comorbidity Survey. Arch Gen Psychiatry.

[3] Maes M, Mylle J, Delmeire L, Janca A (2001). Pre- and post-disaster negative life events in relation to the incidence and severity of post-traumatic stress disorder. Psychiatry Res.

[4] Frans O, Rimmö PA, Aberg L, Fredrikson M (2005). Trauma exposure and post-traumatic stress disorder in the general population. Acta Psychiatr Scand.

[5] Neria Y, DiGrande L, Adams BG (2011). Posttraumatic stress disorder following the September 11, 2001, terrorist attacks: a review of the literature among highly exposed populations. Am Psychol.

[6] Adams RE, Boscarino JA (2006). Predictors of PTSD and delayed PTSD after disaster: The impact of exposure and psychosocial resources. J Nerv Ment Dis.

[7] Pietrzak RH, Feder A, Singh R, Schechter CB, Bromet EJ, Katz CL, Reissman DB, Ozbay F, Sharma V, Crane M, Harrison D, Herbert R, Levin SM, Luft BJ, Moline JM, Stellman JM, Udasin IG, Landrigan PJ, Southwick SM (2014). Trajectories of PTSD risk and resilience in World Trade Center responders: an 8-year prospective cohort study. Psychol Med.

[8] Kline A, Ciccone DS, Weiner M, Interian A, St Hill L, Falca-Dodson M, Black CM, Losonczy M (2013). Gender differences in the risk and protective factors associated with PTSD: a prospective study of National Guard troops deployed to Iraq. Psychiatry.

[9] Horesh D, Solomon Z, Zerach G, Ein-Dor T (2011). Delayed-onset PTSD among war veterans: the role of life events throughout the life cycle. Soc Psychiatry Psychiatr Epidemiol.

[10] Franz MR, Wolf EJ, MacDonald HZ, Marx BP, Proctor SP, Vasterling JJ (2013). Relationships among predeployment risk factors, warzone-threat appraisal, and postdeployment PTSD symptoms. J Trauma Stress.

[11] Tracie Shea M, Reddy MK, Tyrka AR, Sevin E (2013). Risk factors for post-deployment posttraumatic stress disorder in national guard/reserve service members. Psychiatry Res.

[12] DiGangi JA, Gomez D, Mendoza L, Jason LA, Keys CB, Koenen KC (2013). Pretrauma risk factors for posttraumatic stress disorder: a systematic review of the literature. Clin Psychol Rev.

[13] True WR, Rice J, Eisen SA, Heath AC, Goldberg J, Lyons MJ, Nowak J (1993). A twin study of genetic and environmental contributions to liability for posttraumatic stress symptoms. Arch Gen Psychiatry.

[14] Stein MB, Jang KL, Taylor S, Vernon PA, Livesley WJ (2002). Genetic and environmental influences on trauma exposure and posttraumatic stress disorder symptoms: a twin study. Am J Psychiatry.

[15] Afifi TO, Asmundson GJ, Taylor S, Jang KL (2010). The role of genes and environment on trauma exposure and posttraumatic stress disorder symptoms: a review of twin studies. Clin Psychol Rev.

[16] Pitman RK, Rasmusson AM, Koenen KC, Shin LM, Orr SP, Gilbertson MW, Milad MR, Liberzon I (2012). Biological studies of post -traumatic stress disorder. Nat Rev Neurosci.

[17] Skelton K, Ressler KJ, Norrholm SD, Jovanovic T, Bradley-Davino B (2012). PTSD and gene variants: new pathways and new thinking. Neuropharmacology.

[18] Zoladz PR, Diamond DM (2013). Current status on behavioral and biological markers of PTSD: a search for clarity in a conflicting literature. Neurosci Biobehav Rev.

[19] Heim C, Nemeroff CB (2009). Neurobiology of posttraumatic stress disorder. CNS Spectr.

[20] Rohleder N, Joksimovic L, Wolf JM, Kirschbaum C (2004). Hypocortisolism and increased glucocorticoid sensitivity of pro-inflammatory cytokine production in Bosnian war refugees with posttraumatic stress disorder. Biol Psychiatry.

[21] Yehuda R, Boisoneau D, Lowy MT, Giller EL (1995). Dose–response changes in plasma cortisol and lymphocyte glucocorticoid receptors following dexamethasone administration in combat veterans with and without posttraumatic stress disorder. Arch Gen Psychiatry.

[22] Yehuda R (2009). Status of glucocorticoid alterations in posttraumatic stress disorder. Ann N Y Acad Sci.

[23] van Zuiden M, Kavelaars A, Geuze E, Olff M, Heijnen CJ (2013). Predicting PTSD: pre-existing vulnerabilities in glucocorticoid-signaling and implications for preventive interventions. Brain Behav Immun.

[24] van Zuiden M, Geuze E, Willemen HL, Vermetten E, Maas M, Heijnen CJ, Kavelaars A (2011). Pre-existing high glucocorticoid receptor number predicting development of posttraumatic stress symptoms after military deployment. Am J Psychiatry.

[25] van Zuiden M, Kavelaars A, Rademaker AR, Vermetten E, Heijnen CJ, Geuze E (2011). A prospective study on personality and the cortisol awakening response to predict posttraumatic stress symptoms in response to military deployment. J Psychiatr Res.

[26] van Zuiden M, Geuze E, Willemen HL, Vermetten E, Maas M, Amarouchi K, Kavelaars A, Heijnen CJ (2012). Glucocorticoid receptor pathway components predict posttraumatic stress disorder symptom development: a prospective study. Biol Psychiatry.

[27] Niu N, Manickam V, Kalari KR, Moon I, Pelleymounter LL, Eckloff BW, Wieben ED, Schaid DJ, Wang L (2009). Human glucocorticoid receptor alpha gene (NR3C1) pharmacogenomics: gene resequencing and functional genomics. J Clin Endocrinol Metab.

[28] Hauer D, Weis F, Papassotiropoulos A, Schmoeckel M, Beiras-Fernandez A, Lieke J, Kaufmann I, Kirchhoff F, Vogeser M, Roozendaal B, Briegel J, de Quervain D, Schelling G (2011). Relationship ofacommon polymorphism of the glucocorticoid receptor gene to traumatic memories and posttraumatic stress disorder in patients after intensive care therapy. Crit Care Med.

[29] Bachmann AW, Sedgley TL, Jackson RV, Gibson JN, Young RM, Torpy DJ (2005). Glucocorticoid receptor polymorphisms and post-traumatic stress disorder. Psychoneuroendocrinology.

[30] American Psychiatric Association (2013). Diagnostic and Statistical Manual of Mental Disorders.

[31] Huang G, Zhang Y, Momartin S, Cao Y, Zhao L (2006). Prevalence and characteristics of trauma and posttraumatic stress disorder in female prisoners in China. Compr Psychiatry.

[32] Mueser KT, Salyers MP, Rosenberg SD, Ford JD, Fox L, Carty P (2001). Psychometric evaluation of trauma and posttraumatic stress disorder assessments in persons with severe mental illness. Psychol Assess.

[33] Yang DS, Zhang YL, Wang XD, Wang XL, Ma H (1999). Life event scale (LES). Rating Scales for Mental Health.

[34] Peng L, Zhang J, Li M, Li P, Zhang Y, Zuo X, Miao Y, Xu Y (2012). Negative life events and mental health of Chinese medical students: the effect of resilience, personality and social support. Psychiatry Res.

[35] Sun N, Xu Y, Wang Y, Duan H, Wang S, Ren Y, Peng J, Du Q, Shen Y, Xu Q, Zhang K (2008). The combined effect of norepinephrine transporter gene and negative life events in major depression of Chinese Han population. J Neural Transm.

[36] Xiao SY (1994). Social support rating scale: the theoretical basis and research applications. J Clin Psychiatry.

[37] Xie RH, He G, Koszycki D, Walker M, Wen SW (2009). Prenatal social support, postnatal social support, and postpartum depression. Ann Epidemiol.

[38] Kolassa IT, Kolassa S, Ertl V, Papassotiropoulos A, De Quervain DJ (2010). The risk of posttraumatic stress disorder after trauma depends on traumatic load and the catechol-o-methyltransferase Val(158)Met polymorphism. Biol Psychiatry.

[39] Panarelli M, Holloway CD, Fraser R, Connell JM, Ingram MC, Anderson NH, Kenyon CJ (1998). Glucocorticoid receptor polymorphism, skin vasoconstriction, and other metabolic intermediate phenotypes in normal human subjects. J Clin Endocrinol Metab.

[40] Derijk RH (2009). Single nucleotide polymorphisms related to HPA axis reactivity. Neuroimmunomodulation.

[41] Manenschijn L, van den Akker EL, Lamberts SW, van Rossum EF (2009). Clinical features associated with glucocorticoid receptor polymorphisms: an overview. Ann N Y Acad Sci.

[42] Krishnamurthy P, Romagni P, Torvik S, Gold PW, Charney DS, Detera-Wadleigh S, Cizza G (2008). Glucocorticoid receptor gene polymorphisms in premenopausal women with major depression. Horm Metab Res.

[43] Wüst S, Van Rossum EF, Federenko IS, Koper JW, Kumsta R, Hellhammer DH (2004). Common polymorphisms in the glucocorticoid receptor gene are associated with adrenocortical responses to psychosocial stress. J Clin Endocrinol Metab.

[44] Kumsta R, Moser D, Streit F, Koper JW, Meyer J, Wüst S (2009). Characterization of a glucocorticoid receptor gene (GR, NR3C1) promoter polymorphism reveals functionality and extends a haplotype with putative clinical relevance. Am J Med Genet B Neuropsychiatr Genet.

[45] van West D, Van Den Eede F, Del-Favero J, Souery D, Norrback KF, Van Duijn C, Sluijs S, Adolfsson R, Mendlewicz J, Deboutte D, Van Broeckhoven C, Claes S (2006). Glucocorticoid receptor gene-based SNP analysis in patients with recurrent major depression. Neuropsychopharmacology.

[46] van West D, Del-Favero J, Deboutte D, Van Broeckhoven C, Claes S (2010). Associations between common arginine vasopressin 1b receptor and glucocorticoid receptor gene variants and HPA axis responses to psychosocial stress in a child psychiatric population. Psychiatry Res.

[47] Chung CC, Shimmin L, Natarajan S, Hanis CL, Boerwinkle E, Hixson JE (2009). Glucocorticoid receptor gene variant in the 3′ untranslated region is associated with multiple measures of blood pressure. J Clin Endocrinol Metab.

[48] Perlis RH, Adams DH, Fijal B, Sutton VK, Farmen M, Breier A, Houston JP (2010). Genetic association study of treatment response with olanzapine/fluoxetine combination or lamotrigine in bipolar I depression. J Clin Psychiatry.

[49] Hauer D, Kolassa IT, Laubender RP, Mansmann U, Hagl C, Roozendaal B, de Quervain DJ, Schelling G (2013). A genotype-specific, randomized controlled behavioral intervention to improve the neuroemotional outcome of cardiac surgery: study protocol for a randomized controlled trial. Trials.

[50] Velders FP, Dieleman G, Cents RA, Bakermans-Kranenburg MJ, Jaddoe VW, Hofman A, Van Ijzendoorn MH, Verhulst FC, Tiemeier H (2012). Variation in the glucocorticoid receptor gene at rs41423247 moderates the effect of prenatal maternal psychological symptoms on child cortisol reactivity and behavior. Neuropsychopharmacology.

